# Spillover From an Intervention on Antibiotic Prescribing for Family Physicians: A Post Hoc Secondary Analysis of a Randomized Clinical Trial

**DOI:** 10.1001/jamanetworkopen.2025.18261

**Published:** 2025-07-01

**Authors:** Kiran Saqib, Noah Ivers, Kevin A. Brown, Nick Daneman, Valerie Leung, Bradley J. Langford, Gary Garber, Jeremy M. Grimshaw, Michael S. Silverman, Monica Taljaard, Jamie Brehaut, Kednapa Thavorn, Meagan Lacroix, Lindsay Friedman, Jennifer Shuldiner, Tara Gomes, Sharon Gushue, Jerome A. Leis, Merrick Zwarenstein, Kevin L. Schwartz

**Affiliations:** 1 Public Health Ontario, Toronto, Ontario, Canada; 2 ICES, Toronto, Ontario, Canada; 3 Dalla Lana School of Public Health, University of Toronto, Toronto, Ontario, Canada; 4 Li Ka Shing Knowledge Institute, Unity Health Toronto, Toronto, Ontario, Canada; 5 Women’s College Hospital Institute for Health System Solutions and Virtual Care, Women’s College Hospital, Toronto, Ontario, Canada; 6 Institute of Health Policy, Management, and Evaluation, University of Toronto, Toronto, Ontario, Canada; 7 Michael Garron Hospital, Toronto East Health Network, Toronto, Ontario, Canada; 8 Sunnybrook Research Institute, Toronto, Ontario, Canada; 9 Department of Medicine University of Toronto, Toronto, Ontario, Canada; 10 Department of Medicine, University of Ottawa, Ottawa, Ontario, Canada; 11 Clinical Epidemiology Program, Ottawa Hospital Research Institute, Ottawa, Ontario, Canada; 12 School of Epidemiology and Public Health, University of Ottawa, Ottawa, Ontario, Canada; 13 Western University, London, Ontario, Canada; 14 Methodological and Implementation Research Program, Ottawa Hospital Research Institute, Ottawa, Ontario, Canada; 15 School of Epidemiology and Public Health, University of Ottawa, Ottawa, Ontario, Canada; 16 Department of Family and Community Medicine, University of Toronto, Toronto, Ontario, Canada; 17 Leslie Dan Faculty of Pharmacy, University of Toronto, Toronto, Ontario, Canada; 18 Ontario Health, Toronto, Ontario, Canada; 19 Departments of Family Medicine and Epidemiology/Biostatistics, Schulich School of Medicine and Dentistry, Western University, London, Ontario, Canada

## Abstract

**Importance:**

Antibiotic audit and feedback is effective at reducing antibiotic prescribing in primary care.

**Objective:**

To evaluate the spillover of an audit-and-feedback intervention originally targeted at patients aged 65 years or older on a broader population of all age groups.

**Design, Setting, and Participants:**

This is a post hoc secondary analysis of a randomized clinical trial that was conducted among primary care physicians in Ontario, Canada. Physicians were randomized in a 4:1 allocation, with a peer comparison feedback letter sent in January 2022. Physicians in the control group received no letter. The randomized clinical trial was conducted from January 2021 to December 2022, and this analysis was performed from March to June 2024.

**Exposure:**

A mailed antibiotic prescribing feedback letter, with peer comparison.

**Main Outcomes and Measures:**

The primary outcome was the total number of antibiotic prescriptions by physicians at 12 months after the intervention for patients of all ages. This analysis was conducted utilizing a different administrative data source than the original trial; this source contained antibiotic prescription counts for all patient age groups. Data were analyzed with Poisson regression models, adjusted for baseline prescribing and stratified by patient age and sex.

**Results:**

Overall, 4964 of 5097 randomized physicians (97.4%) were included in this analysis. There were 3967 (74.5%) in the intervention group and 997 (25.5%) in the control group; 2766 physicians (55.7%) were male, and 2549 (51.3%) had been in practice for 25 years or more. The intervention group showed a reduction in antibiotic prescriptions at 12 months after intervention compared with the control group (adjusted rate ratio [aRR], 0.93; 95% CI, 0.93-0.94). Significant reductions were seen across all age and sex groups and for antibiotics typically used for respiratory infections. Additionally, the proportion of prescriptions exceeding 7 days decreased significantly in the intervention group (aRR, 0.82; 95% CI, 0.82-0.83).

**Conclusions and Relevance:**

In this post hoc secondary analysis of a randomized clinical trial of peer comparison antibiotic audit and feedback for physicians with data from patients aged 65 and older, the intervention group had a reduction in antibiotic prescriptions across all patient ages. These findings suggest that routinely collected administrative data can be effectively used for implementing and evaluating antibiotic audit and feedback, even when limited to older patients.

**Trial Registration:**

ClinicalTrials.gov Identifier: NCT04594200

## Key Points

**Question:**

Was an antibiotic audit-and-feedback intervention originally targeting prescribing for patients aged 65 years or older associated with spillover to the broader population?

**Findings:**

In this secondary analysis of a randomized clinical trial with 4964 primary care physicians, the intervention group experienced a significant 7% relative reduction in antibiotic prescriptions for patients of all ages compared with the control group at 12 months.

**Meaning:**

In this study, an antibiotic audit-and-feedback intervention for family physicians that focused on antibiotic prescriptions for patients aged 65 years or older was associated with reduced antibiotic prescribing for patients of all ages.

## Teaser

This secondary analysis of a randomized clinical trial examines whether an audit-and-feedback intervention for antibiotic prescribing to patients 65 years and older was associated with prescribing patterns for other age groups.

## Introduction

Antibiotics revolutionized modern medicine, effectively combating bacterial infections, and saving countless lives. However, their overuse and misuse have led to a significant rise in antimicrobial resistance worldwide, posing a grave threat to public health.^1^ The burden of inappropriate antibiotic use in outpatient settings is substantial, contributing to treatment failures, hospital admissions, and the emergence of resistant pathogens.^2,3^

Addressing the complex issue of antimicrobial stewardship has become a public health concern, necessitating the implementation of interventions aimed at optimizing antibiotic use while minimizing the development of resistance. Evidence suggests that implementing outpatient antimicrobial stewardship interventions can reduce inappropriate antibiotic prescribing rates, leading to improved patient outcomes.^3,4,5^

Audit and feedback on antibiotic prescribing is a potentially effective and scalable intervention grounded in behavioral science.^6^ In a recently completed randomized clinical trial (RCT) in Ontario, Canada,^7^ we used administrative data to provide antibiotic prescribing audit and feedback to family physicians, reducing antibiotic prescribing rates by 5%. One important limitation to this study was that the administrative data were limited to prescriptions for patients aged 65 years and older.

Given the limitations of the data in our previous trial, our objective in this study was to conduct a secondary analysis of our recently completed RCT^7^ using a different source of administrative data, which contains antibiotic prescription counts for patients of all ages. We hypothesized that we would find spillover in antibiotic prescribing among the broader patient population.

## Methods

### Design

Originally, we conducted a pragmatic RCT in Ontario, Canada, to evaluate mailed antibiotic audit-and-feedback reports to primary care physicians. Using existing administrative data, we assessed the intervention’s effectiveness. The trial protocol is published^8^ and appears in Supplement 1. This article presents a post hoc secondary analysis of the trial, performed in 2024. In this analysis, we reevaluated the intervention using a different administrative data source that contains antibiotic prescriptions for patients of all ages, treated by the same physicians during the initial RCT. Reporting for this clinical trial followed the Consolidated Standards of Reporting Trials (CONSORT) reporting guideline. Ethics board approvals for the trial were obtained from Women’s College Hospital Research Institute and Public Health Ontario. A waiver of informed consent was granted as the intervention had minimal risks, the trial would not have been practical without the waiver, participants had an opportunity to opt out, and they received a debrief letter at trial completion.^7^

### Participants and Setting

In the original trial,^7^ the enrolled physicians were randomly assigned in a ratio of 4:1 to either receive a peer comparison antibiotic prescribing feedback letter or to not receive any such correspondence. The assignment of physicians into these groups was stratified based on whether they had previously received a similar letter as part of a prior intervention. Those allocated to the intervention group were sent a feedback letter on or after January 15, 2022. An example of the letter is available in the eAppendix in Supplement 2. A follow-up reminder letter, identical to the initial one, was sent approximately 1 month later. The content of the feedback letter included personalized antibiotic prescribing rates with peer comparison as well as the proportion of prescriptions that were for a prolonged duration (defined as >7 days). The letter included educational components on appropriate antibiotic initiation and duration. The letter was designed through iterations of user-centered design, incorporating feedback from physicians and patients aimed at influencing behavioral change to reduce unnecessary antibiotic prescribing as well as prolonged duration prescribing in patients aged 65 years and older. The trial utilized data from the Ontario Drug Benefit database (ODB) that can link prescriber characteristics, patient billing claims, and patient characteristics to prescription data for patients aged 65 years and older. Details of this trial methodology have been published elsewhere.^7,8^ This analysis was not previously planned in this registration.

### Outcomes

The primary outcome for our analysis in this study was antibiotic prescription counts by included physicians for all patients. We further stratified the primary outcome by patient age and sex groups (male patients <18 years, 18-64 years, and ≥65 years and female patients <18 years, 18-64 years, and ≥65 years) as well as by urban vs rural location. The secondary outcomes were the number of prolonged-duration antibiotic prescriptions (defined as >7 days) and the number of respiratory antibiotic prescriptions, including penicillin, cephalosporins, fluoroquinolones, macrolides, and tetracyclines, due to their primary role in treating respiratory infections and their significant contribution to overall antibiotic prescribing practices. We defined more than 7 days as prolonged because most community-managed infections can be effectively treated with a course of antibiotics lasting 7 days or fewer.^9^

### Database

In this study we used the Xponent antibiotic database for all patient ages from IQVIA for our outcomes. This database is a prospectively collected administrative database constructed by directly gathering prescription data from a sample of Ontario pharmacies, accounting for approximately 62% of all dispensed medications. This sample is then projected to represent 100% of all dispensed medications in the province. IQVIA utilizes a patented geospatial projection algorithm, which incorporates additional sales and insurance claims data as well as geographical information from pharmacies not initially captured, to produce the projected data. Although the specific methodology utilized by IQVIA is proprietary, the data has been internally validated by IQVIA. A previous external validation study from Ontario found that the dataset can accurately identify high-prescribing physicians, with some discrepancy between urban and rural settings.^10^

We linked the original trial data cohort with the database for aggregated dispensed antibiotic prescription counts at the level of individual physician prescribers on an annual basis. The prescription counts excluded refills, topical, and intravenous antibiotics.

### Statistical Analysis

The randomization groups and the trial dataset were linked to the database using College of Physician and Surgeons of Ontario (CPSO) numbers. The baseline period was defined as the calendar year 2021 (January 1 to December 31), with the calendar year 2022 (January 1 to December 31) as the intervention period. This post hoc secondary analysis was performed from March to June 2024. The association of the intervention with total antibiotic prescription counts was evaluated using a similar prespecified analytical approach used in the previously published trial. We analyzed the number of antibiotic prescriptions at the level of the physician, using Poisson regression, adjusted for the log of the baseline antibiotic prescription counts, physician sex, years since medical graduation, and stratification variable of whether the physician had previously received antibiotic prescribing feedback, similar to our previous trial analysis.^7^ The notable difference in this study is that the data utilized did not include a patient visit denominator, and the outcome in this study was antibiotic prescription counts (rather than antibiotic prescribing rates in the original trial analysis).

We repeated the analysis for all antibiotics stratifying patients into 6 groups: male patients younger than 18 years, female patients younger than 18 years, male patients aged 18 to 64 years, female patients aged 18 to 64 years, male patients aged 65 years or older, and female patients aged 65 years or older. We then repeated the analysis limiting to respiratory antibiotic counts, which included penicillins (penicillin, amoxicillin, cloxacillin, and amoxicillin-clavulinic acid), second- and third-generation cephalosporins (cefuroxime, cefprozil, cefoxitin, and cefixime), third-generation fluoroquinolones (levofloxacin and moxifloxacin), macrolides (erythromycin, clarithromycin, and azithromycin), and tetracyclines (tetracycline and doxycycline) due to their primary role in treating respiratory infections and their significant contribution to overall antibiotic prescribing practices. Additionally, we assessed the association of the intervention arm with the proportion of antibiotic prescriptions exceeding 7 days in duration (prolonged duration) for all patients and in each demographic group. Given our prior validation study indicating potentially reduced data validity in rural areas, we conducted a stratified analysis of the primary outcome based on the geographical location of physicians (rural vs urban areas). We also conducted a stratified analysis by physician baseline prescribing volume using approximate tertiles (low, medium, and high). Intervention effect sizes were expressed using adjusted rate ratios (aRRs) and 95% CIs. The data were analyzed using SAS EG version 8.2 (SAS Institute). *P* < .05 was considered statistically significant.

## Results

**Figure 1.**
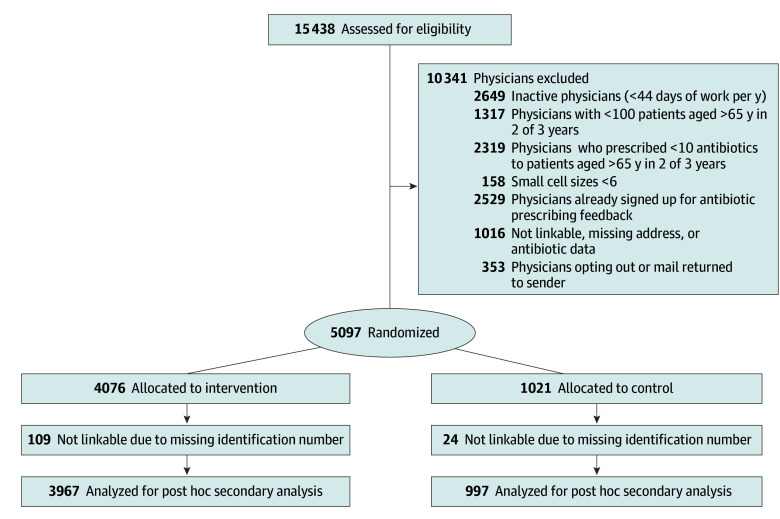
Study Flowchart The diagram of study inclusion and exclusion criteria was modified from the original trial.^8^

**Figure 2.**
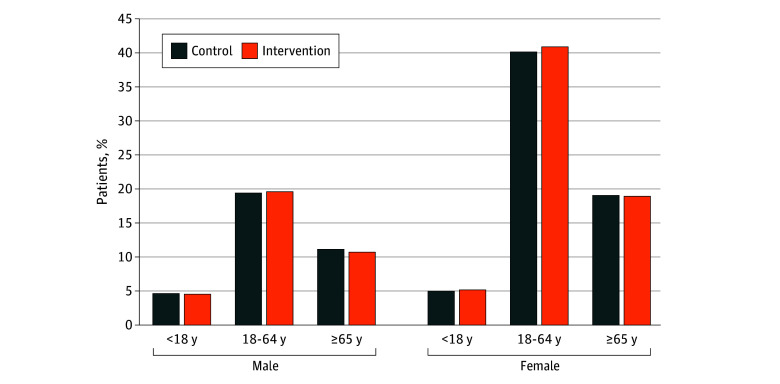
Proportion of Patients Prescribed Antibiotics by Physicians in the Intervention and Control Groups During the Study Period, by Patient Age and Sex

In the original trial, 5097 physicians were randomized and mailed the intervention. For this study, after excluding 133 physicians due to missing CPSO identification number, 4964 physicians (97.4%) were included, with 3967 (74.5%) in the intervention group and 997 (25.5%) in the control group (Figure 1). More than half of physicians were male (2766 [55.7%]), and 2549 (51.3%) were in practice for 25 years or more (Table 1). Antibiotics were prescribed by study physicians most frequently to female patients aged 18 to 64 years, followed by male patients aged 18 to 64 years and female patients 65 years or older (Figure 2).

**Table 1.** Baseline Characteristics of Physicians

Characteristic	Physicians, No. (%)		
	Total	Control	Intervention
Total (row %)	4964 (100)	997 (20.1)	3967 (79.9)
Sex			
Female	2198 (44.3)	420 (42.1)	1778 (44.8)
Male	2766 (55.7)	577 (57.8)	2189 (55.1)
Years in practice			
1-10	908 (18.3)	176 (17.6)	732 (18.4)
11-24	1507 (30.4)	299 (29.9)	1208 (30.4)
≥25	2549 (51.3)	522 (52.3)	2027 (51.1)
Geography			
Urban	4559 (91.8)	912 (91.4)	3647 (91.9)
Rural	405 (8.2)	85 (8.5)	320 (8.0)

**Figure 3.**
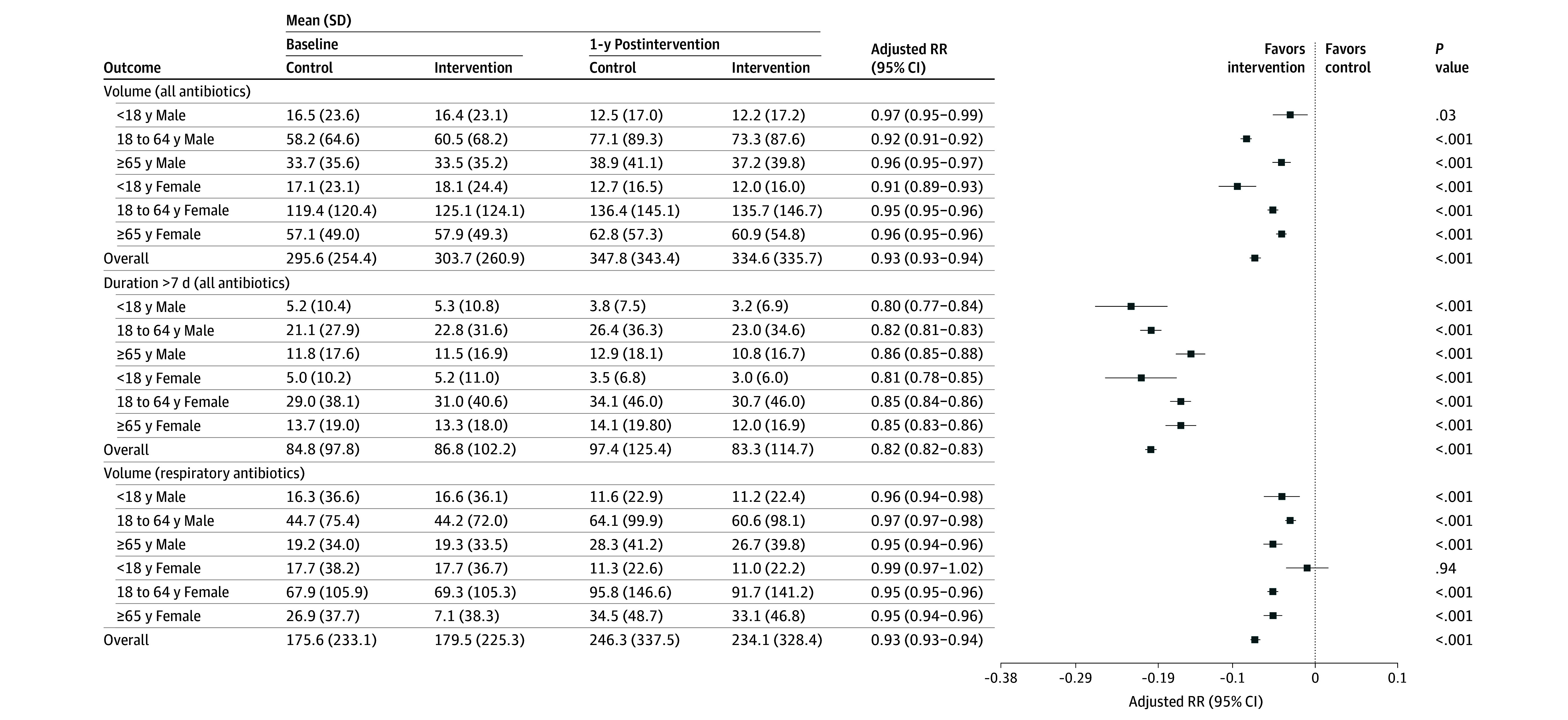
Comparison of Antibiotic Prescribing Outcomes for Physicians in Intervention vs Control Groups at 12 Months After the Intervention, Stratified by Patient Age and Sex RR indicates relative risk.

In the baseline year, the mean (SD) antibiotic prescription count was 303.7 (260.9) in the intervention group and 295.6 (254.4) in the control group. At 12 months after the intervention, the mean (SD) antibiotic prescription count was 334.6 (335.7) in the intervention group and 347.8 (343.4) in the control group. At the 12-month follow-up, the intervention group had significantly lower overall antibiotic prescriptions counts compared with the control group with an aRR of 0.93 (95% CI, 0.93- 0.94) (Figure 3).

In the analysis stratified by patient age and sex groups, the intervention was associated with a significant decrease in antibiotic prescriptions at the 12-month interval among patients aged 65 years and older, both male and female, similar to the original trial results. The intervention was also associated with significantly reduced antibiotic prescriptions in several other groups: female patients younger than 18 years (aRR, 0.91; 95% CI, 0.89-0.93), male patients aged 18 to 64 years (aRR, 0.92; 95% CI, 0.91-0.92), and female patients aged 18 to 64 years (aRR, 0.95; 95% CI, 0.95-0.96). Furthermore, we observed a significant decrease in the number of prescriptions exceeding 7 days in duration within the intervention group across all age groups (aRR, 0.82; 95% CI, 0.82-0.83). This reduction in prolonged prescriptions was evident across all age groups, including both male and female patients younger than 18 years, those aged 18 to 64 years, and those 65 years and older. Similar results were observed in the sensitivity analysis, which was limited to respiratory antibiotics only (Figure 3).

Two different subgroup analyses were conducted to further understand the outcomes associated with the intervention. First, we conducted a sensitivity analysis stratifying the cohort by the geographical location of physicians, comparing antibiotic use in urban vs rural areas. Urban and rural areas had aRRs of 0.93 (95% CI, 0.93-0.94) and 0.98 (95% CI, 0.97-0.99), respectively, for antibiotic use. Second, we divided physicians into 3 groups based on baseline antibiotic prescribing counts. The results showed similar reductions across these categories, with aRRs of 0.93 (95% CI, 0.92-0.94) for low, 0.97 (95% CI, 0.96-0.97) for medium, and aRR, 0.95 (95% CI, 0.94-0.95) for high prescribing physicians (Table 2).

**Table 2.** Subgroup Analysis of Antibiotic Prescribing Outcomes for Intervention vs Control at 12 Months After the Intervention

Outcome	Mean (SD)				Adjusted RR (95%CI)		*P* value	
	Baseline		1 y					
	Control	Intervention	Control	Intervention				
**Geographical location**							
Urban	89.9 (149.6)	92.9 (154.1)	104.1 (190.4)	100.7 (185.7)	0.93 (0.93-0.94)	<.001	
Rural	77.8 (128.3)	72.8 (122.8)	87.8 (152.1)	80.8 (144.0)	0.98 (0.97-0.99)	<.001	
**Baseline antibiotic prescribing counts**							
Low	101.9 (44.6)	103.7 (44.9)	127.1 (109.4)	118.5 (96.9)	0.93 (0.92-0.94)	<.001	
Medium	252.9 (45.8)	256.2 (46.0)	291.7 (184.5)	282.5 (173.4)	0.97 (0.96-0.97)	<.001	
High	661.5 (239.9)	676.6 (239.1)	775.5 (375.3)	738.5 (383.6)	0.95 (0.94-0.95)	<.001	

Abbreviation: RR, rate ratio.

## Discussion

This study evaluated potential spillover from an RCT of family physician antibiotic prescribing feedback reports that resulted in reduced antibiotic use for older adults to assess whether this finding was seen across all age groups. In the previous study we observed a 4% relative reduction in antibiotic prescribing rates at 12 months (aRR, 0.96; 95% CI, 0.95-0.97) and a 14% relative reduction in prolonged duration prescribing (aRR, 0.86; 95% CI, 0.85-0.87) among adults 65 years or older.^7^ In this secondary analysis of that trial including all age groups, we observed similar findings, with a 7% relative reduction in antibiotic prescription counts and 18% relative reduction in prolonged duration prescription counts. The effect sizes were consistent across various age and sex groups, underscoring the intervention’s outcomes for older adults and spillover to the broader population, including younger age groups.

The spillover of feedback interventions for antibiotic prescribing practices can be explained by several potential mechanisms. One primary mechanism is the universal relevance of the messaging and education presented in the feedback letters. Although the feedback might be derived from data specific to a subgroup, such as patients aged 65 years and older, the principles for responsible antibiotic use are applicable across all age groups. This broad applicability ensures that the data resonates with physicians’ overall practice patterns, rather than being limited to a specific patient demographic. This is supported by the findings of recent studies^11,12,13^ that observed a reduction in antibiotic prescribing across various age groups following an intervention aimed at promoting responsible antibiotic use in primary care settings. Previous research^14,15^ has indicated that audit-and-feedback interventions can effectively modify physician behavior and reduce unnecessary antibiotic use. A systematic review and meta-analysis^16^ found that audit and feedback generally lead to small but potentially important improvements in professional practice, with a median absolute increase in compliance with desired practice of 4.3%. Specific to antibiotic prescribing, research^17^ shows that socially motivated interventions, including peer comparison, significantly reduce inappropriate antibiotic prescriptions among primary care physicians. This is largely attributed to the competitive and normative pressures it generates among physicians, who are inclined to align their practices with those of their peers to avoid being perceived as outliers.

The audit-and-feedback approach aims to optimize the effectiveness of antimicrobial stewardship programs. Studies^18,19,20^ have shown that combining audit and feedback with educational and communication components can significantly improve antibiotic prescribing practices and overall antimicrobial use. Prolonged duration prescribing in antimicrobial treatments is often due to gaps in knowledge among prescribers, which can be effectively addressed through educational interventions. We have previously demonstrated that a simple intervention, such as mailing a table with guidelines, significantly improved prescribing practices.^21^

Our study found that audit-and-feedback interventions using administrative data for older patients was associated with a broader shift in prescribing behavior, promoting judicious antibiotic use across all age groups. These findings have important implications for jurisdictions like our own that do not have readily available access to antibiotic prescribing data for the entire population. We have demonstrated that despite this limitation in data, antibiotic feedback reports passively delivered to a large population of primary care physicians broadly impacts antibiotic prescribing behaviors. This comprehensive impact addresses antibiotic resistance, a major public health concern, by reducing overprescribing—a key contributor to resistance. By influencing prescribing behavior beyond the initially targeted group, these interventions support the scalability and generalizability of antimicrobial stewardship programs using routinely collected administrative data. This practice change, while small, likely improves overall care quality, reduces antibiotic-related adverse events, and prevents the development of resistant bacterial strains. The spillover effect of these interventions, internalized by physicians and applied universally, highlights their potential to drive systemic improvements in health care quality and patient outcomes. We believe that antibiotic audit and feedback programs should be incorporated into all primary care antimicrobial stewardship programs.

Future research should explore the long-term sustainability of the intervention’s associations with antibiotic prescribing. Additionally, investigating the underlying mechanisms driving behavior change in response to peer comparison audit and feedback could provide valuable insights for refining and optimizing such interventions. Studies could also examine the impact of similar interventions in different health care settings and among different specialties to assess their generalizability and effectiveness across various contexts. Audit-and-feedback interventions should be evaluated for both positive and negative spillover effects to capture the full breadth of impact of these interventions for health care systems. Furthermore, comparing the approaches between adult and pediatric populations could offer important distinctions and strategies tailored to different age groups.

### Limitations

This study has some notable limitations. The data used in this analysis included antibiotic prescription counts opposed to rates denominated per patient visit as was used in the original trial, which may limit the comparability of the analysis between studies. However, despite the lack of denominator data, the results appeared quite comparable. The data are based on projected data. While we have previously validated these data in Ontario, errors are possible, in particular in rural settings, which may have explained the discrepancy observed between urban and rural areas.^10^ While these data are derived from the use of statistically representative samples and data imputation methods, they do not capture a census of activity. Moreover, due to the proprietary nature of the source’s data imputation methods, they cannot be published in this literature; however, IQVIA does provide documentation and training on such methods to licensees of their data. The data used are based on antibiotic dispensing from pharmacies and not directly antibiotic consumption by patients. We could not evaluate for intervention outcomes beyond 12 months in this study because both the intervention and control groups received antibiotic prescribing feedback at 12 months in Ontario. The focus on prescribing counts does not account for clinical appropriateness or patient outcomes. Furthermore, while we observed a clinical and statistically significant association between the intervention and the outcomes, the overall effect size was small, highlighting the importance of additional research to improve audit and feedback and cointerventions to support antimicrobial stewardship.

## Conclusions

In this post hoc secondary analysis of an RCT of peer comparison antibiotic audit and feedback for physicians with data from patients aged 65 years and older, the intervention group had a reduction in antibiotic prescriptions across all patient ages. This finding underscores the potential for spillover effects of such interventions to contribute significantly to efforts aimed at reducing inappropriate antibiotic prescribing and antibiotic resistance. By fostering more judicious prescribing practices among family physicians, peer comparison audit-and-feedback interventions could enhance patient care quality and support public health objectives.

## Notes

Supplement 1.
Trial Protocol

Supplement 2. **eAppendix.** Example Peer Letter

Supplement 3.
Data Sharing Statement
