# Supplementary material for: Spillover From an Intervention on Antibiotic Prescribing for Family Physicians: A Post Hoc Secondary Analysis of a Randomized Clinical Trial
Source: JAMA Netw Open. 2025 Jul 1;8(7):e2518261. doi: 10.1001/jamanetworkopen.2025.18261 (PMC12215572; doi:10.1001/jamanetworkopen.2025.18261)
Supplement: Supplement 1. — Trial Protocol [file jamanetwopen-e2518261-s001.pdf]

Re-evaluation of a randomized controlled trial of peer comparison audit and feedback on family physician antibiotic prescribing to older adults: assessing impacts on younger patients

Background:

Combating rising antimicrobial resistance (AMR) is an urgent public health priority according to the Public Health Agency of Canada and the World Health Organization. Antibiotic overuse is strongly correlated with rising drug resistance, and antibiotic stewardship programs are necessary to curb overprescribing. Reducing unnecessary antibiotic use is an important quality improvement initiative which will improve patient care, reduce antibiotic adverse events, reduce healthcare utilization, and slow AMR. One component of antimicrobial stewardship that has demonstrated effectiveness is peer comparison audit and feedback; whereby antibiotic prescribing is measured and provided back to physicians compared to their peers. These interventions are rooted in behavioural science and we have demonstrated in two sequential randomized controlled trials (RCTs) in Ontario that they significant reduce total and inappropriate antibiotic use.

The initial trial was done using IQVIA data which included all patients and told high-prescribing physicians they had a higher number of antibiotic prescriptions than 75% of their peers. These data lack clinical information and cannot account for practice size.(Schwartz et al. 2021) Our second trial included all physicians and utilized the Ontario Drug Benefit (ODB) database within ICES administrative data which is highly accurate and linkable to other administrative data, however it is limited to patients 65 years of age and older. In this trial, analyzed using ODB data, we demonstrated significant reductions in overall antibiotic prescribing rates to patients 65+ (RR 0.95; 95%CI, 0.94-0.96).(Shuldiner et al. 2022)

We have previously demonstrated a strong correlation between antibiotic prescribing to patients 65+ years and total patient populations at the physician level (Spearman correlation coefficient 0.80 for male and 0.84 for female patients). In the previous trial the feedback and evaluation were limited to patient 65+ years, however we hypothesize that the intervention had similar effect sizes in younger patient age groups. Our objective for this study is to conduct a secondary analysis of this study using a different data source that includes antibiotics prescribed to all patients to determine the impact of the audit and feedback intervention on the general population.

Methods:

We propose a secondary analysis of a recently completed RCT, that was previously analyzed using ODB data that included only patients 65+ years, using IQVIA data and all patient age groups. The methods for the previous trial have been published.(Shuldiner et al. 2022) Physicians were randomized 4:1 to receive a peer comparison antibiotic prescribing feedback letter or not. Physician groups were stratified based on whether they have received a previous letter or not from our previous intervention. Physicians randomized to receive the intervention were mailed one letter on or after January 15 2022. The same letter was sent again as a reminder approximately 2 months later. The controls did not receive any letter. The letter contained their antibiotic prescribing rate compared to the 25th percentile of their peers with behavioural change messaging on reducing unnecessary antibiotic prescribing. The letter contained guidance on antibiotic durations, communications training, and links to resources from

Choosing Wisely Canada. The letter was co-signed from the Chief Medical Officer of Health in Ontario, the president of the Ontario College of Family Practice, and the chair from Choosing Wisely Canada.

Database:

The IQVIA Xponent database contains dispensed antibiotic prescription counts aggregated to the physician prescriber-level per year. Antibiotics are grouped as total and into 13 classes, and includes only outpatient antibiotic prescriptions dispensed from a pharmacy. Prescriptions counts exclude refills, topical, and intravenous medications. The database also includes antibiotic prescribing rates (antibiotic prescriptions per 100 total prescriptions), proportion of prescriptions for various durations, and is further stratified by patient age and sex (males <18 years, females <18 years, males 18-64 years, females 18-64 years, males 65+ years, females 65+ years). IQVIA creates the Xponent database by obtaining prescription data directly from a proportion of Ontario pharmacies (~50%); equating to ~60% of dispensed medications. IQVIA then incorporates sales and insurance claims data, as well as geographical location of pharmacies not captured, into a patented geospatial projection algorithm which extrapolates the data to all physician prescribed medications in Ontario. The methodology is proprietary, but internally validated by IQVIA. We have also previously externally validated the Xponent dataset compared to the ODB at ICES and demonstrated that the data is accurate.(Schwartz et al. 2019) Xponent contains variables of CPSO number, physician demographics and address, years since medical graduation, physician sex, and chronic disease score which is a patient comorbidity index aggregated to the physician level.

Analysis:

We will link the randomization arms, and stratification variable, to Xponent using CPSO numbers. The baseline period will be defined as calendar year 2021 and the intervention year 2022. We will evaluate the effect of the intervention on total antibiotic prescription counts. We will use Poisson regression models adjusting for the log of the baseline antibiotic prescription counts, sex, years since medical graduation, and stratification variable. We will repeat this analysis for each patient age and sex group. We will repeat the analysis for the proportion of antibiotic prescriptions that are >7 days (prolonged duration) for all patients and each patient age and sex group.

Due to our previous validation study demonstrating reduced accuracy in rural areas we will conduct a stratified analysis of the primary outcome by rural/urban location using the second digit of the Forward Sortation Area (FSA); 0=rural. We will also perform a subgroup analysis of respiratory antibiotics only (penicillins, penicillin+beta lactamase inhibitors, macrolides, 3rd generation fluoroquinolones, and 2nd/3rd generation cephalosporins).

Ethical/privacy considerations:

The completed trial has ethics review board approval from Public Health Ontario. All data is currently held within PHO including randomization arms, CPSO numbers, and the IQVIA databases.

Anticipated impacts:

This analysis will provide important information on the generalizability of our feedback interventions on the general population beyond patients 65 years of age and older. The findings will be used to support

ongoing antibiotic prescribing feedback in Ontario as well as other jurisdictions lacking full population level prescribing data.

Limitations:

The antibiotic counts are aggregated into 20 equal physicians. Therefore, we will use the mean antibiotic count for the group as a proxy for the physicians' actual antibiotic prescription counts. However, the strength is we can evaluate by 6 different age and sex groupings, antibiotic prescribing rates, as well as prolonged duration prescribing proportions.

References:

Schwartz, Kevin L., Cynthia Chen, Bradley J. Langford, Kevin A. Brown, Nick Daneman, Jennie Johnstone, Julie H. C. Wu, Valerie Leung, and Gary Garber. 2019. "Validating a Popular Outpatient Antibiotic Database to Reliably Identify High Prescribing Physicians for Patients 65 Years of Age and Older." *PloS One* 14 (9): e0223097.

Schwartz, Kevin L., Noah Ivers, Bradley J. Langford, Monica Taljaard, Drew Neish, Kevin A. Brown, Valerie Leung, et al. 2021. "Effect of Antibiotic-Prescribing Feedback to High-Volume Primary Care Physicians on Number of Antibiotic Prescriptions: A Randomized Clinical Trial." *JAMA Internal Medicine* 181 (9): 1165–73.

Shuldiner, Jennifer, Kevin L. Schwartz, Bradley J. Langford, and Noah M. Ivers. 2022. "Optimizing Responsiveness to Feedback about Antibiotic Prescribing in Primary Care: Protocol for Two Interrelated Randomized Implementation Trials with Embedded Process Evaluations." *Implementation Science: IS* 17 (1): 1–17.
