# Supplementary material for: Spillover From an Intervention on Antibiotic Prescribing for Family Physicians: A Post Hoc Secondary Analysis of a Randomized Clinical Trial
Source: JAMA Netw Open. 2025 Jul 1;8(7):e2518261. doi: 10.1001/jamanetworkopen.2025.18261 (PMC12215572; doi:10.1001/jamanetworkopen.2025.18261)
Supplement: Supplement 2. — eAppendix. Example Peer Letter [file jamanetwopen-e2518261-s002.pdf]

Supplemental Online Content

Saqib K, Ivers N, Brown KA, et al. Spillover from an intervention on antibiotic prescribing for family physicians. *JAMA Netw Open.* 2025;8(7):e2518261. doi:10.1001/jamanetworkopen.2025.18261

eAppendix. Example Peer Letter

This supplemental material has been provided by the authors to give readers additional information about their work.

© 2025 Saqib K et al. *JAMA Network Open*.

eAppendix. Example Peer Letter

Attention Dr. Jon Smith

December, 2021

Public Health Ontario, Choosing Wisely Canada, and the Ontario College of Family Physicians are partnering to help you optimize your antibiotic prescribing. We are providing family physicians across Ontario their **confidential prescribing data** along with evidence-based recommendations on antibiotic prescribing.

Your personal antibiotic prescribing data

From March 2020-February 2021, **80% of your peers prescribed fewer antibiotics than you did.**

Around 25-50% of antibiotics prescribed by the average Ontario family physician are unnecessary[1,2]. One quarter of your peers are prescribing XX or fewer antibiotics per 1000 visits and this may be an achievable target for you. We understand that there are many factors that influence prescribing; however, the data suggest an opportunity for you to change your prescribing.

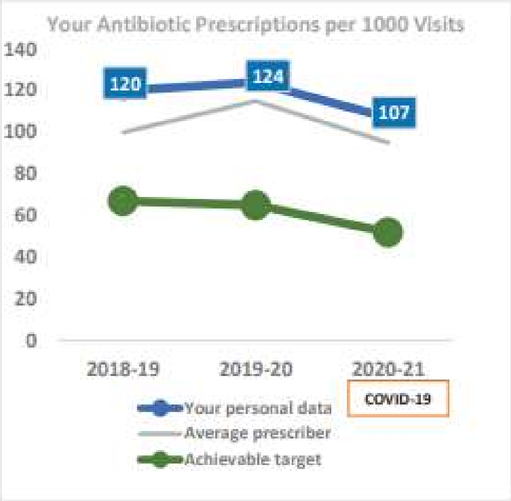

We recognize in the era of COVID-19, primary care clinics adopted virtual care and are experiencing challenges regarding how to manage respiratory tract infections including when to test, when to prescribe antibiotics, and when to see a patient in-person. Also, there are challenges such as diagnostic uncertainty, patient expectations, and time constraints. The goal is not to eliminate antibiotic prescribing, but to reduce unnecessary prescriptions.

© 2025 Saqib K et al. *JAMA Network Open*.

Antibiotics cause patient harms

Patients may be more willing to forego antibiotics when they hear that 30% of primary care patients will experience side effects.[3]

Think twice before prescribing antibiotics for respiratory tract infections

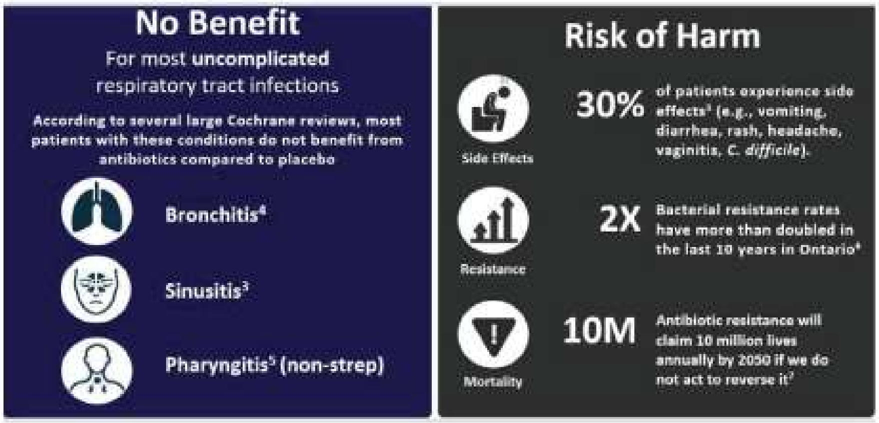

Already, **about 26% of bacterial infections in Canadians are resistant to first line antibiotics** and by the year 2050, this rate is expected to be 40%.[8]

- An estimated 25% of primary care antibiotic prescriptions in Ontario are unnecessary.[1]
- Antibiotic prescriptions that are unnecessary are not associated with better patient outcomes. Your antibiotic prescribing may lead to antimicrobial resistance.
- The COVID-19 pandemic has led to more virtual care, but should not lead to unnecessary antibiotic prescribing.

Collect MAINPRO+ credits

This letter may be used to complete a Linking Learning. Simply login to the Mainpro portal (portal.cfpc.ca), select "Assessment," "Certified" and "Linking learning to assessment" and complete the form.

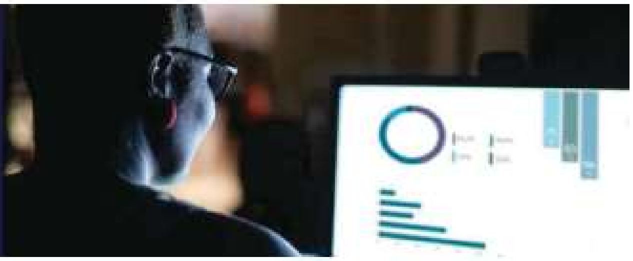

© 2025 Saqib K et al. *JAMA Network Open*.

Shorter is better

If you must prescribe, keep in mind that most community-acquired infections should be treated for ≤ 7 days. Long durations of treatment in primary practice may be necessary for complicated infections, such as osteomyelitis, endocarditis or deep undrained abscesses.

34% of your antibiotic prescriptions were for >7 days duration.

Recommended duration for antibiotics based on latest evidence

Syndrome	Evidence-based-recommended duration*
Acute sinusitis	5 days
Pneumonia	5 days
Cellulitis	5 days
Otitis Media	5 days (10 days in children <2 years)
Cystitis	3-5 days (depending on drug)
Acute exacerbation of COPD	5 days

**References: see page 7. Reassessment is important to ensure patients are following the expected course. These recommended durations assume clinical improvement at this time point.*

© 2025 Saqib K et al. *JAMA Network Open*.

Communication tips

Most patients do not expect antibiotics – they want to feel better. Here are some ways to talk to those patients who are asking for antibiotics.[9]

If your patient says...	Evidence-based communication tips[10]*
I always get antibiotics for my bronchitis... it worked last time	**State your examination findings:** I can see you are not feeling well, *Virtual Visit*: however, based on what you have told me / *In-person visit*: but my examination of your throat, ears and chest was normal.<br>**State your diagnosis:** I understand this may feel similar to bronchitis you may have experienced before, but it is more likely that your chest cold right now is caused by a virus and not bacteria.<br>**Provide recommendations:** The latest research tells us that antibiotics will not help you get better faster and could cause side-effects. The best way to treat a chest cold is with plenty of rest and fluids. You can also try honey to relieve coughing or lozenges for throat pain. You can ease pain and reduce fever with Acetaminophen or Ibuprofen.<br>**Create a follow-up plan:** A chest cold typically last 1-3 weeks. If your illness takes longer or if you are worsening, we should arrange a follow-up visit.

Communicating these key messages in this order, including negative (e.g., you do not need antibiotics)* ***and* *positive (e.g., honey to relieve cough) recommendations has been demonstrated to reduce unnecessary antibiotic use, improve patient satisfaction, and reduce clinic visit time*[10]

Antibiotic prescribing, virtual care, and COVID-19

In the era of COVID-19, virtual care is common. Fortunately, the majority of acute respiratory tract infections (RTIs) are viral and can be managed via virtual visits. For those RTIs that may be bacterial, a physical examination is recommended to confirm the diagnosis (e.g., to assess the tympanic membrane or to perform a Strep test) prior to prescribing an antibiotic. If you frequently prescribe antibiotics for RTIs after virtual assessments alone, you may be overprescribing.

Scan here to learn more.

© 2025 Saqib K et al. *JAMA Network Open*.

Public Health Ontario | Santé publique Ontario   Ontario College of Family Physicians   Choosing Wisely Ontario

Viral prescription pads

Enclosed is a viral prescription pad. You can also easily integrate it into your EMR in several languages: http://bit.ly/abx-prescriptions

There are a number of ways to provide a patient with a viral prescription virtually:

- Verbally review the viral prescription with your patient.
- Fill it in and email it directly to your patient (if it is in your EMR).
- Scan/take a photo of it and email it to your patient

Scan here to learn more.   Choosing Wisely Ontario

The pad, e-forms and accompanying instructions were co-developed by OntarioMD, Choosing Wisely Canada, Rx Files and the CFPC.

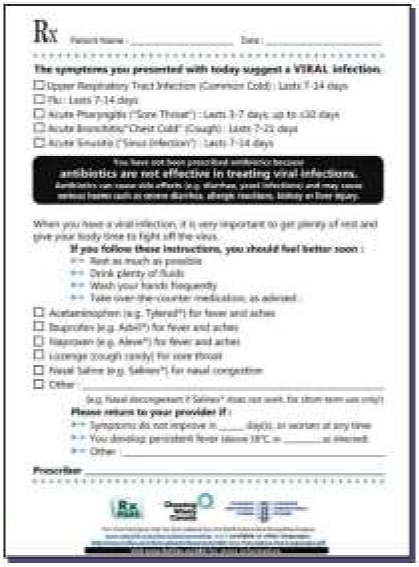
Rx Patient Name: ______ Date: ______

The symptoms you presented with today suggest a VIRAL infection.

- ☐ Upper Respiratory Tract Infection (Common Cold) : Lasts 7-14 days
- ☐ Flu : Lasts 7-14 days
- ☐ Acute Pharyngitis ("Sore Throat") : Lasts 3-7 days; up to ≤10 days
- ☐ Acute Bronchitis/"Chest Cold" (Cough) : Lasts 7-21 days
- ☐ Acute Sinusitis ("Sinus Infection") : Lasts 7-14 days

You have not been prescribed antibiotics because
antibiotics are not effective in treating viral infections.
[illegible]

When you have a viral infection, it is very important to get plenty of rest and give your body time to fight off the virus.

If you follow these instructions, you should feel better soon :

- Rest as much as possible
- Drink plenty of fluids
- Wash your hands frequently
- Take over-the-counter medication, as advised :

- ☐ Acetaminophen (e.g. Tylenol®) for fever and aches
- ☐ Ibuprofen (e.g. Advil®) for fever and aches
- ☐ Naproxen (e.g. Aleve®) for fever and aches
- ☐ Lozenge (cough candy) for sore throat
- ☐ Nasal Saline (e.g. Salinex®) for nasal congestion
- ☐ Other : ______

(e.g. Nasal decongestant if Salinex® does not work, for short-term use only)

Please return to your provider if :

- Symptoms do not improve in ____ day(s), or worsen at any time
- You develop persistent fever (above 38°C or ______ as directed)
- Other : ______

Prescriber ______

Are you interested in receiving feedback electronically? You may be eligible for Ontario Health (Quality) MyPractice reports. They are confidential documents that give family physicians an informative perspective of their individual practice. To learn more, visit their website at www.hqontario.ca/pc-sign-up.

You can find Choosing Wisely materials that can help with antibiotic prescribing at http://bit.ly/abx-pc.

For questions about this report or to opt out of future reports, you may contact us at asp@oahpp.ca.

Sincerely,

Dr. Kieran Moore MD FRCPC<br>Ontario Chief<br>Medical Officer of Health	Dr. Jennifer Young MD CFPC<br>Past president<br>Ontario College of Family Physicians	Dr. Wendy Levinson MD<br>Chair<br>Choosing Wisely Canada

© 2025 Saqib K et al. *JAMA Network Open*.

Additional information about this letter

Where did we get these data?

These data are meant for your own quality improvement. We cannot share it with anyone except you. The data for this report were derived from the Ontario Drug Benefit (ODB) database housed at ICES (formerly, the Institute for Clinical Evaluative Sciences). The ODB database captures >99% of dispensed prescriptions, but is limited to patients ≥ 65 years of age; **however, antibiotic prescribing in patients ≥ 65 is highly correlated with overall antibiotic use for all age groups among Ontario family physicians.** We have also provided data on peer comparison to give context to the findings. Physicians were excluded from the intervention if they saw less than 100 patients ≥ 65 years of age or prescribed less than 10 antibiotics to patients ≥ 65 years of age in two of the three years of data.

How did we define your peers for comparison?

Total antibiotic prescribing was defined as the total number of oral antibiotic prescriptions written by yourself, to a patient ≥ 65 years of age, that were dispensed by an outpatient pharmacy. Your prescribing rate was adjusted for patient volume. Your peers were defined as other family physicians in Ontario.

Data were obtained from ICES (using a combination of OHIP billings and hospital discharge data). Physicians with the lowest prescribing quartile represent an achievable target for many family physicians in Ontario. As a society, we overuse antibiotics, particularly for respiratory tract infections. For context, in Sweden, physicians prescribe about half the number of antibiotics per population.[11] Approximately one quarter of antibiotics prescribed by primary care physicians in Ontario are unnecessary.[1] We encourage you to use the tools provided in this letter, as well as your data, to reflect on your antibiotic prescribing.

What is the evidence for shorter antibiotic durations?

Numerous randomized controlled trials have been performed for common infectious diseases that have consistently showed that shorter courses of antibiotics are non-inferior to standard or longer courses. This does not mean that all patients can be treated with shorter durations and consideration should be given to the type of drug used, comorbidities and the patient's response to therapy. Follow-up to ensure improvement and expected resolution is important. The studies have been nicely summarized at https://www.bradspellberg.com/shorter-is-better.

© 2025 Saqib K et al. *JAMA Network Open*.

Example references used to guide duration recommendations are:

Sinusitis: Falagas M E, et al. Effectiveness and safety of short vs. long duration of antibiotic therapy for acute bacterial sinusitis: a meta-analysis of randomized trials. *British Journal of Clinical Pharmacology*. 2009 Feb; 67(2):161-71.

Pneumonia: Uranga A, et al. Duration of Antibiotic Treatment in Community-Acquired Pneumonia A Multicenter Randomized Clinical Trial. *JAMA Intern Med.* 2016, 176(9): 1257-1265.

Cellulitis: Moran G J, et al. Tedizolid for 6 days versus linezolid for 10 days for acute bacterial skin and skin-structure infections (ESTABLISH-2): a randomised, double-blind, phase 3, non-inferiority trial. *The Lancet Infectious Diseases*. 2014, 14(8): 696-705.

Otitis Media: Hoberman A, et al. Shortened antimicrobial treatment for acute otitis media in young children. *N Engl J Med.* 2016, 375: 2446-56.

Cystitis: Kim D K, et al. Reappraisal of the treatment duration of antibiotic regimens for acute uncomplicated cystitis in adult women: a systematic review and network meta-analysis of 61 randomised clinical trials. *The Lancet Infectious Diseases*. 2020, 20(9): 1080-1088.

COPD Exacerbation: Stolbrink M, et al. Does antibiotic treatment duration affect the outcomes of exacerbations of asthma and COPD? A systematic review. *Chronic Respiratory Disease*. 2018, 15(3): 225-40.

References

1. Schwartz K L, et al. Unnecessary antibiotic prescribing in a Canadian primary care setting: a descriptive analysis using routinely collected electronic medical record data. *CMAJ Open*. 2020, 8(2):E360-E369.
2. Silverman M, et al. Antibiotic prescribing for nonbacterial acute upper respiratory infections in elderly persons. *Annals of Internal Medicine*. 2017, 166(11): 765-774.
3. Lemiengre M B, et al. Antibiotics for acute rhinosinusitis in adults. *Cochrane Database Systematic Reviews*. 2018 Sep 10; 9(9).
4. Smith S M, et al. Antibiotics for acute bronchitis. *Cochrane Database of Systematic Reviews*. 2017(6).
5. Spinks A, et al. Antibiotics for sore throat. *Cochrane Database of Systematic Reviews*. 2013(11).
6. Toronto Invasive Bacterial Diseases Network. Amoxicillin resistance in respiratory isolates. Accessed October 1, 2021. http://www.tibdn.ca/data-publications/data/streptococcus-pneumoniae/amoxicillin-resistance-respiratory
7. O'Neill J. Antimicrobial resistance: tackling a crisis for the health and wealth of nations. *Review on Antimicrobial Resistance.* December, 2014.
8. Council of Canadian Academies. When Antibiotics Fail: The Expert Panel on the Potential Socio-Economic Impacts of Antimicrobial Resistance in Canada. Ottawa (ON), November 12, 2019. Accessed October 1, 2021. https://cca-reports.ca/reports/the-potential-socio-economic-impacts-of-antimicrobial-resistance-in-canada/
9. Kianmehr H, et al. Patient expectation trends on receiving antibiotic prescriptions for respiratory tract infections: A systematic review and meta-regression analysis. *International Journal of Clinical Practice*. 2019, 73(7): e13360.
10. Mangione-Smith R, et al. Communication practices and antibiotic use for acute respiratory tract infections in children. *The Annals of Family Medicine*. 2015, 13(3): 221-227.
11. Mölstad S, et al. Lessons learnt during 20 years of the Swedish strategic programme against antibiotic resistance. *Bulletin of the World Health Organization*. 2017, 95(11): 764.

© 2025 Saqib K et al. *JAMA Network Open*.
