# Supplementary material for: Spillover From an Intervention on Antibiotic Prescribing for Family Physicians: A Post Hoc Secondary Analysis of a Randomized Clinical Trial
Source: JAMA Netw Open. 2025 Jul 1;8(7):e2518261. doi: 10.1001/jamanetworkopen.2025.18261 (PMC12215572; doi:10.1001/jamanetworkopen.2025.18261)
Supplement: Supplement 3. — Data Sharing Statement [file jamanetwopen-e2518261-s003.pdf]

Data Sharing Statement

Saqib. Spillover From an Intervention on Antibiotic Prescribing for Family Physicians. *JAMA Netw Open*. Published July 01, 2025. doi:10.1001/jamanetworkopen.2025.18261

Data
Additional Information: Trial Registration: NCT04594200
https://clinicaltrials.gov/study/NCT04594200
Data available: No

Additional Information
Explanation for why data not available: Data Availability Statement: The data for this study are not publicly available. The IQVIA Xponent dataset is owned by and proprietary to IQVIA. The license agreement between PHO and IQVIA does not permit us to share the data publicly. The authors had no special access privileges and other researchers may license the data from IQVIA directly (www.iqvia.com).
